# Supplementary material for: Which cues are sexy? The evolution of mate preference in sympatric species reveals the contrasted effect of adaptation and reproductive interference
Source: Evol Lett. 2024 Jan 2;8(2):283–94. doi: 10.1093/evlett/qrad058 (PMC10959492; doi:10.1093/evlett/qrad058)
Supplement: qrad058_suppl_Supplementary_Appendix [file qrad058_suppl_supplementary_appendix.pdf]

Appendix: Which cues are sexy? The evolution of mate preference in sympatric species reveals the contrasted effect of adaptation and reproductive interference.

Ludovic Maisonneuve[1,2,*], Charline Smadi[3], Violaine Llaurens[1]

1. Institut de Systématique, Evolution, Biodiversité (ISYEB), Muséum national d'Histoire naturelle, CNRS, Sorbonne Université, EPHE, Université des Antilles CP 50, 57 rue Cuvier, 75005 Paris, France;

2. Department of Ecology and Evolution, University of Lausanne, 1004 Lausanne, Switzerland;

3. Univ. Grenoble Alpes, INRAE, LESSEM, F-38402 St-Martin-d'Hères, France and Univ. Grenoble Alpes, CNRS, Institut Fourier, 38610 Gières, France;

∗ Corresponding author; e-mail: ludovic.maisonneuve.2015@polytechnique.org.

A1 QLE analysis

Evolution of mating traits under natural and sexual selection

First, we explored the relative effects of natural and sexual selections on the evolution of traits in species A. Following the QLE approach, the change of allele 1 frequency at T_i, for $i \in \{1,2\}$, after one generation in this species is given by:

$$\Delta P_{T_i} = G_{T_i} \overbrace{\left(s_i + \overline{\rho}_i \left(P_{P_i} - \frac{1}{2}\right)\right)}^{\text{natural and sexual selections}} \overbrace{-2u_{T_i}\left(P_{T_i} - \frac{1}{2}\right)}^{\text{action of mutations}} + O(\varepsilon^2), \tag{A1}$$

where G_I is the genetic diversity at locus $I \in \{T_1, T_2, P_1, P_2, M\}$ given by

$$G_I = P_I(1 - P_I), \tag{A2}$$

and $\overline{\rho}_1$ and $\overline{\rho}_2$ are the average strengths of preference on traits T_1 and T_2 respectively in the population

$$\overline{\rho}_1 = \rho\left((1 - P_M)f(1 - \gamma_{\mathrm{r}}) + P_M f(1 - \gamma_{\mathrm{m}})\right), \tag{A3}$$

$$\overline{\rho}_2 = \rho\left((1 - P_M)f(\gamma_{\mathrm{r}}) + P_M f(\gamma_{\mathrm{m}})\right). \tag{A4}$$

While the action of natural selection simply depends on the advantage of trait value 1 due to natural selection s_i, the effect of sexual selection is modulated by the average strength of preference on trait T_i in the population ($\overline{\rho}_i$). Sexual selection promotes the allele 1 when most females prefer the associated trait value 1 *i.e.* when $P_{P_i} > 1/2$.

Evolution of trait value preference

Now we explore the selection acting on preference loci P_1 and P_2, determining which trait value is sexy at traits T_1 and T_2. Following the QLE approach, the change of allele 1 frequency at P_i, for $i \in \{1,2\}$, after one generation in this species is given by:

$$\Delta P_{P_i} = \Delta^{\text{dir-RI}} P_{P_i} + \Delta^{\text{dir-c}} P_{P_i} + \Delta^{\text{ind}} P_{P_i} - 2u_{P_i}\left(P_{P_i} - \frac{1}{2}\right), \tag{A5}$$

where $\Delta^{\text{dir-RI}} P_{P_i}$, $\Delta^{\text{dir-c}} P_{P_i}$, $\Delta^{\text{ind}} P_{P_i}$ and $-2u_{P_i}(P_{P_i} - 1/2)$ describes the effect of direct selection due to reproductive interference and cost of choosiness, indirect selection and mutations.

Reproductive interference promotes preference for the trait value more common within conspecific than heterospecific.

The effect of reproductive interference on the change of allele 1 frequency at P_i, for $i \in \{1,2\}$ is given by

$$\Delta^{\text{dir-RI}} P_{P_i} = G_{P_i} \overline{\rho}_i c_{\mathrm{ri}} \frac{\tilde{N}}{N}(P_{T_i} - \tilde{P}_{T_i}), \tag{A6}$$

where $\tilde{P}_{T_i}$ is the frequency of allele 1 at locus T_i in species B.

Selection acting on locus P_i depends on how much preference targets the trait T_i, captured by $\overline{\rho}_i c_{\mathrm{ri}}$. Reproductive interference promotes preference for the trait value 1 when trait value 1 is more common within conspecific than within heterospecific *i.e.* $P_{T_i} - \tilde{P}_{T_i}$.

As expected, the effect of reproductive interference mainly depends on the density ratio between species B and A, $\tilde{N}/N$: the probability that a female in species A encounters an heterospecific male increases with $\tilde{N}/N$. Selection caused by reproductive interference also increases with the strength of preference ρ. Strong preference leads to more significant fitness differences between females, with different preferences intensifying selection due to reproductive interference.

Sympatry with other species intensifies the cost of choosiness

Preference allows the rejection of heterospecific males but also leads to the rejection of conspecific males. After rejecting a male, a female has a probability c of not encountering another male leading to an opportunity cost. The effect of these costs on the change of allele 1 frequency at P_i, for $i \in \{1, 2\}$ is given by

$$\Delta^{\text{dir-c}} P_{P_i} = G_{P_i} \overline{\rho}_i c \frac{N + \tilde{N}}{N} \left(P_{T_i} - \frac{1}{2} \right). \tag{A7}$$

The cost of choosiness disfavours preference for trait value 1 when this trait value is the scarcest in the population *i.e.* $P_{T_i} < 1/2$. Surprisingly, selection due to cost of choosiness increases with the proportion of heterospecifics. When a female rejects a conspecific male, she must wait for another suitable male. However, females will be likely to encounter heterospecific males before encountering a conspecific male, making the rejection of a conspecific more dramatic when conspecific males are rare. The effect of the cost of choosiness is thus proportional to the average number of males a female will encounter until she encounters a conspecific $(N + \tilde{N})/N$.

Indirect selection promotes preference producing locally adapted offspring and sexy sons

Frequencies at preference loci P_1 and P_2 not only directly change the fitness because this modifies reproductive interference and the cost of choosiness but also because of associations with different alleles at the traits loci T_1 and T_2 in the offspring, leading to contrasted indirect fitness benefits. Within offspring, the preference allele at locus P_i becomes associated with the preferred alleles at trait T_i for $i \in \{1, 2\}$. Under the QLE assumptions, the genetic association between alleles 1 at loci T_i and P_i, for $i \in \{1, 2\}$, is given by

$$D_{T_i P_i} = \overline{\rho}_i G_{T_i} G_{P_i}. \tag{A8}$$

Because of mate preference, there is a positive association between the genetic basis of preference for one trait value and the genetic basis of this trait value.

The term describing the effect of indirect selection on the change of allele 1 frequency at P_i, for $i \in \{1, 2\}$ is given by

$$\Delta^{\text{ind}} P_{P_i} = D_{T_i P_i} \left(s_i + \overline{\rho}_i \left(P_{P_i} - \frac{1}{2} \right) \right). \tag{A9}$$

When the mutant is associated with a trait value, direct selection on this trait indirectly affects the change of mutant frequency. Then indirect selection promotes the evolution of preference towards trait value promoted by natural or sexual selection.

Evolution of a mutant modifying *relative preference weighting*

We investigate the evolution of the focus of female preference on either trait. We thus study the invasion of a mutant at locus M associated with the value γ_{m}, differing from the value γ_{r} associated with the ancestral allele. Under the QLE approximation, the allele frequency variation at the preference locus can be divided into three terms, denoted $\Delta^{\text{dir-RI}}, \Delta^{\text{dir-c}}$ and $\Delta^{\text{ind}} P_M$, reflecting the effect of direct selection due to reproductive interference and the cost of choosiness and indirect selection, on the change of the mutant frequency ΔP_M respectively.

$$\Delta P_M = \Delta^{\text{dir-RI}} P_M + \Delta^{\text{dir-c}} P_M + \Delta^{\text{ind}} P_M + O(\varepsilon^3). \tag{A10}$$

Reproductive interference promotes preference targeting the trait leading to strongest heterospecific avoidance.

The effect of reproductive interference on the change of mutant frequency is given by

$$\begin{aligned} \Delta^{\text{dir-RI}} P_M = G_M c_{\text{ri}} \frac{\tilde{N}}{N} \Bigg(& \delta\rho_1 \left(P_{P_1} - \frac{1}{2} \right) \left(P_{T_1} - \tilde{P}_{T_1} \right) \\ & + \delta\rho_2 \left(P_{P_2} - \frac{1}{2} \right) \left(P_{T_2} - \tilde{P}_{T_2} \right) \Bigg), \end{aligned} \tag{A11}$$

where $\delta\rho_1$ and $\delta\rho_2$ quantify the effect of the mutant allele on the preference for trait T_1 and T_2, respectively, compared to the resident allele:

$$\delta\rho_1 = \rho \left(f(1-\gamma_{\mathrm{m}}) - f(1-\gamma_{\mathrm{r}}) \right), \tag{A12}$$

$$\delta\rho_2 = \rho \left(f(\gamma_{\mathrm{m}}) - f(\gamma_{\mathrm{r}}) \right). \tag{A13}$$

For instance, when $\delta\rho_2 > 0$, the mutant allele leads to more attention on trait T_2 than the resident allele. Note that f is an increasing function: $\delta\rho_1$ and $\delta\rho_2$ thus have opposite signs, *i.e.* when mutant allele increases female attention on one trait, it also decreases female attention on the other trait.

Reproductive interference promotes preference for the trait allowing more accurate species recognition. Selection due to reproductive interference depends on relative phenotypic frequencies in both species. Preference for a trait leads to increased intraspecific matings than expected under random mating when the targeted trait is more common within species A than within species B. The higher the difference in trait frequencies between species, the more substantial species recognition is.

Because we have

$$\delta\rho_1 \approx -\delta\gamma\rho h'(1-\gamma_{\mathrm{r}}), \tag{A14}$$

$$\delta\rho_2 \approx \delta\gamma\rho h'(\gamma_{\mathrm{r}}), \tag{A15}$$

the term S_{ri} describing the effect of reproductive interference on mutant fitness is given by

$$\begin{aligned} S_{\text{ri}} = c_{\text{ri}} \frac{\tilde{N}}{N} \rho \Bigg(& - h'(1-\gamma_{\mathrm{r}}) \left(P_{P_1} - \frac{1}{2} \right) \left(P_{T_1} - \tilde{P}_{T_1} \right) \\ & + h'(\gamma_{\mathrm{r}}) \left(P_{P_2} - \frac{1}{2} \right) \left(P_{T_2} - \tilde{P}_{T_2} \right) \Bigg). \end{aligned} \tag{A16}$$

The cost of choosiness affects mutant fate

The effect of the cost of choosiness on mutant frequency change is given by

$$\begin{aligned}\Delta^{\text{dir-c}} P_M = &- G_M \frac{c}{2}\frac{N+\tilde{N}}{N}\Big(\delta\rho_1\left(P_{P_1}(1-P_{T_1}) + (1-P_{P_1})P_{T_1}\right)\\ &+ \delta\rho_2\left(P_{P_2}(1-P_{T_2}) + (1-P_{P_2})P_{T_2}\right)\Big).\end{aligned} \tag{A17}$$

The fate of a mutant depends on the match at each trait between the most preferred trait value and the most common trait value.

The term S_c describing the effect of cost of choosiness on mutant fitness is thus given by

$$\begin{aligned}S_\text{c} = &- \frac{c}{2}\frac{N+\tilde{N}}{N}\rho\Big(-h'(1-\gamma_\text{r})\left(P_{P_1}(1-P_{T_1}) + (1-P_{P_1})P_{T_1}\right)\\ &+ h'(\gamma_\text{r})\left(P_{P_2}(1-P_{T_2}) + (1-P_{P_2})P_{T_2}\right)\Big).\end{aligned} \tag{A18}$$

Indirect selection promotes preference on the trait providing the most substantial indirect fitness benefit

The term describing the effect of indirect selection on mutant alleles at locus M is given by

$$\begin{aligned}\Delta^{\text{ind}} P_M = &\overbrace{D_{T_1M}}^{\substack{\text{genetic association}\\ \text{between } T_1 \text{ and } M}} \overbrace{\left(s_1 + \overline{\rho}_1\left(P_{P_1} - \frac{1}{2}\right)\right)}^{\text{direct selection on } T_1} \\ &+ \underbrace{D_{T_2M}}_{\substack{\text{genetic association}\\ \text{between } T_2 \text{ and } M}} \underbrace{\left(s_2 + \overline{\rho}_2\left(P_{P_2} - \frac{1}{2}\right)\right)}_{\text{direct selection on } T_2},\end{aligned} \tag{A19}$$

where D_{T_1M} (resp. D_{T_2M}) is the genetic association between the mutant allele at locus M and allele 1 at locus T_1 (resp. T_2), see (A20). When the mutant is associated with a trait value, direct selection on this trait indirectly affects the change of mutant frequency.

The genetic association between the mutant at locus M and the trait T_i, for $i \in \{1,2\}$, is given by

$$D_{T_iM} = G_{T_i}G_M\delta\rho_i\left(P_{P_i} - \frac{1}{2}\right) + O(\varepsilon^2). \tag{A20}$$

When the mutant leads to more attention on T_i ($\delta\rho_i > 0$), the mutant becomes associated with the allele with the most preferred trait value at T_i. Accordingly, when the mutant leads to less attention on T_i ($\delta\rho_i < 0$), it is associated with the allele with the least preferred trait value at T_i.

The terms S_os and S_or describing the effect of offspring survival and reproductive success on mutant fitness are thus given by

$$S_\text{os} = \rho\Big(-G_{T_1}h'(1-\gamma_\text{r})\left(P_{P_1} - \frac{1}{2}\right)s_1 + G_{T_2}h'(\gamma_\text{r})\left(P_{P_2} - \frac{1}{2}\right)s_2\Big), \tag{A21}$$

and

$$S_{\mathrm{or}} = \rho^2 \Bigg(- G_{T_1} h'(1-\gamma_{\mathrm{r}}) \left(P_{P_1} - \frac{1}{2} \right) f(1-\gamma_{\mathrm{r}})(P_{P_1} - \frac{1}{2}) + G_{T_2} h'(\gamma_{\mathrm{r}}) \left(P_{P_2} - \frac{1}{2} \right) f(\gamma_{\mathrm{r}})(P_{P_2} - \frac{1}{2}) \Bigg). \tag{A22}$$

A2 Equilibrium allele frequencies at traits and preference loci T_1, T_2, P_1 and P_2

In an adaptive dynamics framework, we study the invasion of a rare mutant at locus M associated with the value of *relative preference weighting* γ_{m} in a resident population where the resident allele codes for the value γ_{r}. Before the mutant introduction, allele frequencies at traits and preference loci T_1, T_2, P_1 and P_2 evolve toward equilibrium allelic frequencies values named $P^*_{T_1}$, $P^*_{T_2}$, $P^*_{P_1}$ and $P^*_{P_2}$. At these equilibrium frequencies, we have

$$\Delta P_{T_1} = \Delta P_{T_2} = \Delta P_{P_1} = \Delta P_{P_2} = 0. \tag{A23}$$

Note we may also have (A23) for different frequencies, so the reached allelic frequencies at loci T_1, T_2, P_1 and P_2 ($P^*_{T_1}$, $P^*_{T_2}$, $P^*_{P_1}$ and $P^*_{P_2}$) may depend on the initial frequencies at these loci.

The changes of allele frequencies at loci T_1 and P_1 (resp. at loci T_2 and P_2) do not depend on the allele frequency at loci T_2 and P_2 (resp. at loci T_1 and P_1) (see Equations (A1) and (A5)). So that before the mutant introduction, allele frequencies at loci T_1 and P_1 coevolve independently of allele frequencies at loci T_2 and P_2. We first explain how to get the value of $P^*_{T_1}$ and $P^*_{P_1}$ without focusing on loci T_2 and P_2. $P^*_{T_1}$ and $P^*_{P_1}$ depend on the coevolution of trait and preference, only happening when resident females weight preference on trait T_1. We then discriminate two cases depending on whether or not females are weighting preference on trait T_1.

Case 1: resident females weight no preference on trait T_1 ($\gamma_{\mathrm{r}} = 1$)

We first compute the equilibrium allelic frequencies $P^*_{T_1}$ and $P^*_{P_1}$ when resident females weight no preference on trait T_1 ($\gamma_{\mathrm{r}} = 1$). In this particular case, no selection acts at the locus P_1. Because of the effect of symmetric mutations (see Equation (A5)) we have

$$P^*_{P_1} = \frac{1}{2}. \tag{A24}$$

We now investigate the equilibrium allele frequencies at locus T_1. Injecting $P^*_{P_1} = 1/2$ in the equation $\Delta P_{T_1} = 0$ gives

$$P^*_{T_1}(1 - P^*_{T_1}) s_1 - 2u_{T_1} \left(P^*_{T_1} - \frac{1}{2} \right) = 0, \tag{A25}$$

which admits a unique solution in the interval $[0, 1]$ given by

$$P^*_{T_1} = \frac{s_1 - 2u_{T_1} + \sqrt{s_1^2 + 4u^2_{T_1}}}{2s_1}. \tag{A26}$$

Importantly the equilibrium allele frequencies $P^*_{T_1}$ and $P^*_{P_1}$ never depend on the initial allele frequency at loci T_1 and P_1.

Case 2: resident females weight preference on trait T_1 ($\gamma_r < 1$)

We now compute the equilibrium allelic frequencies $P^*_{T_1}$ and $P^*_{P_1}$ when resident females weight preference on trait T_1 ($\gamma_r < 1$). To find candidate value of $P^*_{T_1}$ and $P^*_{P_1}$ we resolve the system of equations $\Delta P_{T_1} = \Delta P_{P_1} = 0$. According to the equation $\Delta P_{T_1} = 0$, $P^*_{T_1}$ and $P^*_{P_1}$ verify

$$P^*_{T_1}(1 - P^*_{T_1})\left(s_1 + \rho f(1-\gamma_r)\left(P^*_{P_1} - \frac{1}{2}\right)\right) - 2u_{T_i}\left(P_{T_i} - \frac{1}{2}\right) = 0, \tag{A27}$$

which gives

$$P^*_{P_1} = \frac{P^*_{T_1}(1 - P^*_{T_1})\left(\rho f(1-\gamma_r) - 2s_1\right) + 4\left(P^*_{T_1} - \frac{1}{2}\right)u_{T_1}}{2P^*_{T_1}(1 - P^*_{T_1})\rho f(1-\gamma_r)}. \tag{A28}$$

Note $P^*_{P_1} \neq 0$ and $P^*_{P_1} \neq 1$ because $\Delta P_{T_1} = u_{T_i} > 0$ when $P_{T_1} = 0$ and $\Delta P_{T_1} = -u_{T_i} < 0$ when $P_{T_1} = 1$ (see Equation (A1)). This ensures that Equation (A28) is always well defined.

Injecting (A28) into (A5) we find that $P^*_{T_1}$ verifies (see Mathematica file in the GitHub repository)

$$Q(P^*_{T_1}) = 0, \tag{A29}$$

where Q is a polynomial function of degree 5, whose exact expression is provided in the Mathematica file.

$P^*_{T_1}$ is one of the roots of the polynomial Q. In our model exploration, we numerically estimate the roots of Q within $[0,1]$ giving all the candidate values for $P^*_{T_1}$. Using Equation (A28) we compute the corresponding candidate values for $P^*_{P_1}$. We then study the convergence stability of each pair of candidate values for $P^*_{T_1}$ and $P^*_{P_1}$. Then depending on the initial allele frequencies at loci T_1 and P_1, we determine the equilibrium allele frequencies reached before the mutant introduction.

We use a similar procedure to compute the values of $P^*_{T_2}$ and $P^*_{P_2}$.

Dependency on initial allele frequencies at loci T_1, T_2, P_1 and P_2

The resident population's equilibrium allele frequencies at loci T_1, T_2, P_1, and P_2 may depend on the initial allele frequencies at these loci. Here we test this dependency for all parameter values used in our article and for all resident *relative preference weighting* values γ_r. The equilibrium allele frequencies in the resident population are independent of the initial allele frequencies for all resident *relative preference weighting* values γ_r when

1. For all γ_r in $[0,1]$, there is a unique tuple of candidate values for $P^*_{T_1}$, $P^*_{P_1}$, $P^*_{T_2}$ and $P^*_{P_2}$.
2. This unique tuple of candidate values is convergence stable.

It appears that in the parameters space explored in this study, the equilibrium allele frequencies of the resident population are always independent of the initial allele frequencies for all resident *relative preference weighting* value γ_r (see Figure A1).

A3 Table and Figures

Abbreviation	Description
$\mathcal{L}$	Set of different loci: $\mathcal{L} = \{T_1, P_1, T_2, P_2, M\}$.
$\mathcal{G}$	Set of different genotypes: $\mathcal{G} = \{0,1\}^5$
$f_i/\tilde{f}_i$	Frequency of genotype i in species A or B.
$P_I/\tilde{P}_I$	Frequency of allele 1 at locus I, for $I \in \mathcal{L}$, in species A or B.
$N/\tilde{N}$	Density of species A/B.
G_I	Genetic diversity at locus I in species A: $G_I = P_I(1 - P_I)$ for $I \in \mathcal{L}$.
$(I)_i$	Allele at locus I of the genotype i for $(I,i) \in \mathcal{L} \times \mathcal{G}$.
$D_{\mathcal{I}}$	Genetic association between alleles at loci in $\mathcal{I}$: $D_{\mathcal{I}} = \sum_{i \in \mathcal{G}} f_i \prod_{I \in \mathcal{I}} (P_I - (I)_i)$ for $\mathcal{I} \subset \mathcal{L}$.
$f'_i/f''_i/f'''_i$	Frequency of genotype i in species a after natural selection/reproduction/mutation.
s_n	Selective advantage of allele 1 at locus T_n, $n \in \{1,2\}$.
γ	*Relative preference weighting.*
$\gamma_{\mathrm{r}}/\gamma_{\mathrm{m}}$	*Relative preference weighting* associated with the resident/mutant allele.
$\gamma_{\mathrm{t_0}}$	Ancestral *relative preference weighting.*
f	Trade-off function determining the relative focus of females on either trait displayed by males.
a	Trade-off parameter tuning the shape of the function f
ρ	Strength of female preference.
c_{ri}	Strength of reproductive interference.
c	Cost of choosiness.
u_I	Mutation rate at locus $I \in \mathcal{L}$.

Table A1: Description of variables and parameters used in the model.

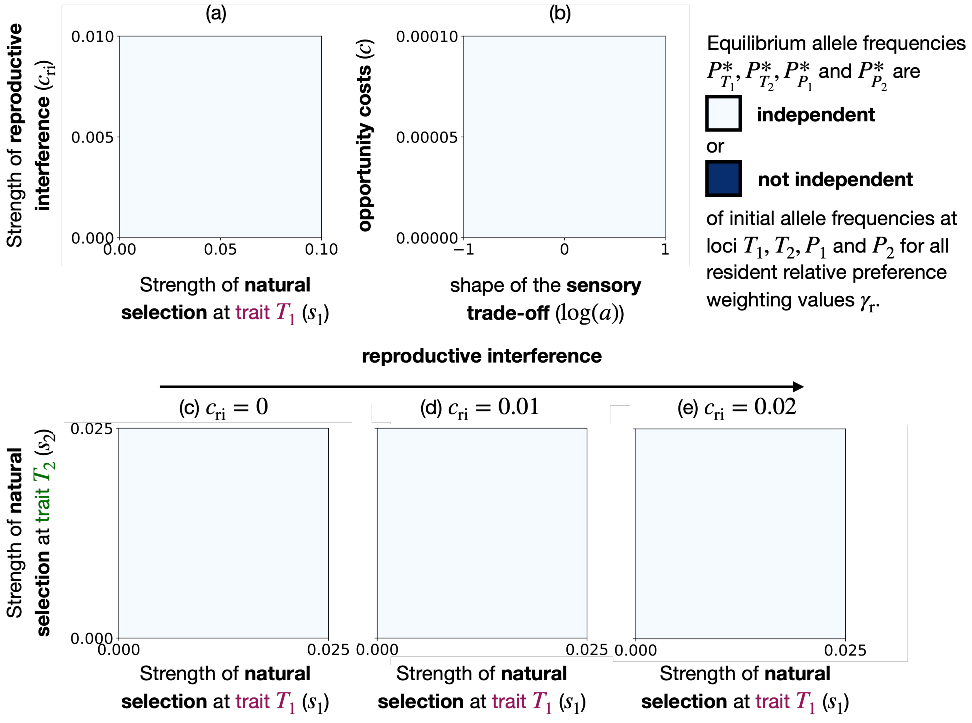

Figure A1: **Equilibrium allele frequencies at loci T_1, T_2, P_1 and P_2 in the resident population is always independent of the initial allele frequencies at these loci for all resident *relative preference weighting* values γ_r.** Parameters values same as (a) Figure 3, (b) Figure 5, (c) Figure 6(a), (d) Figure 6(b) and (e) Figure 6(c).

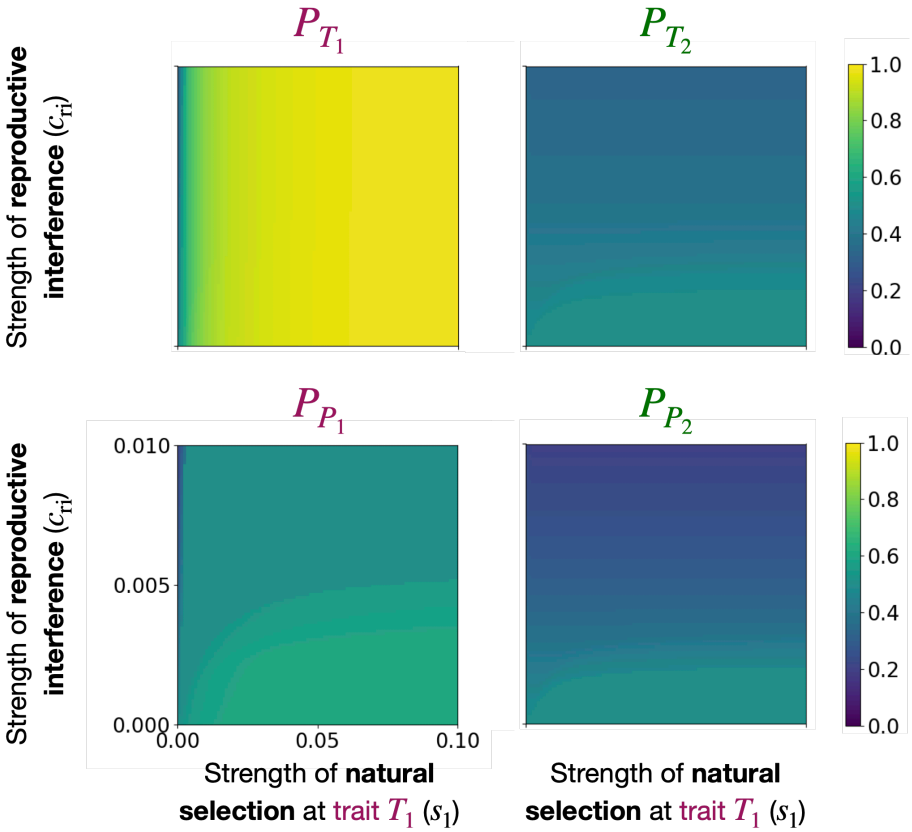

Figure A2: **Frequency of allele 1 at loci T_1, T_2, P_1 and P_2 at equilibrium preference ($\gamma_r = \gamma^*$), depending on the strength of natural selection acting on trait T_1 (s_1) and the strength of reproductive interference (c_{ri}), when T_2 is neutral ($s_2 = 0$).**

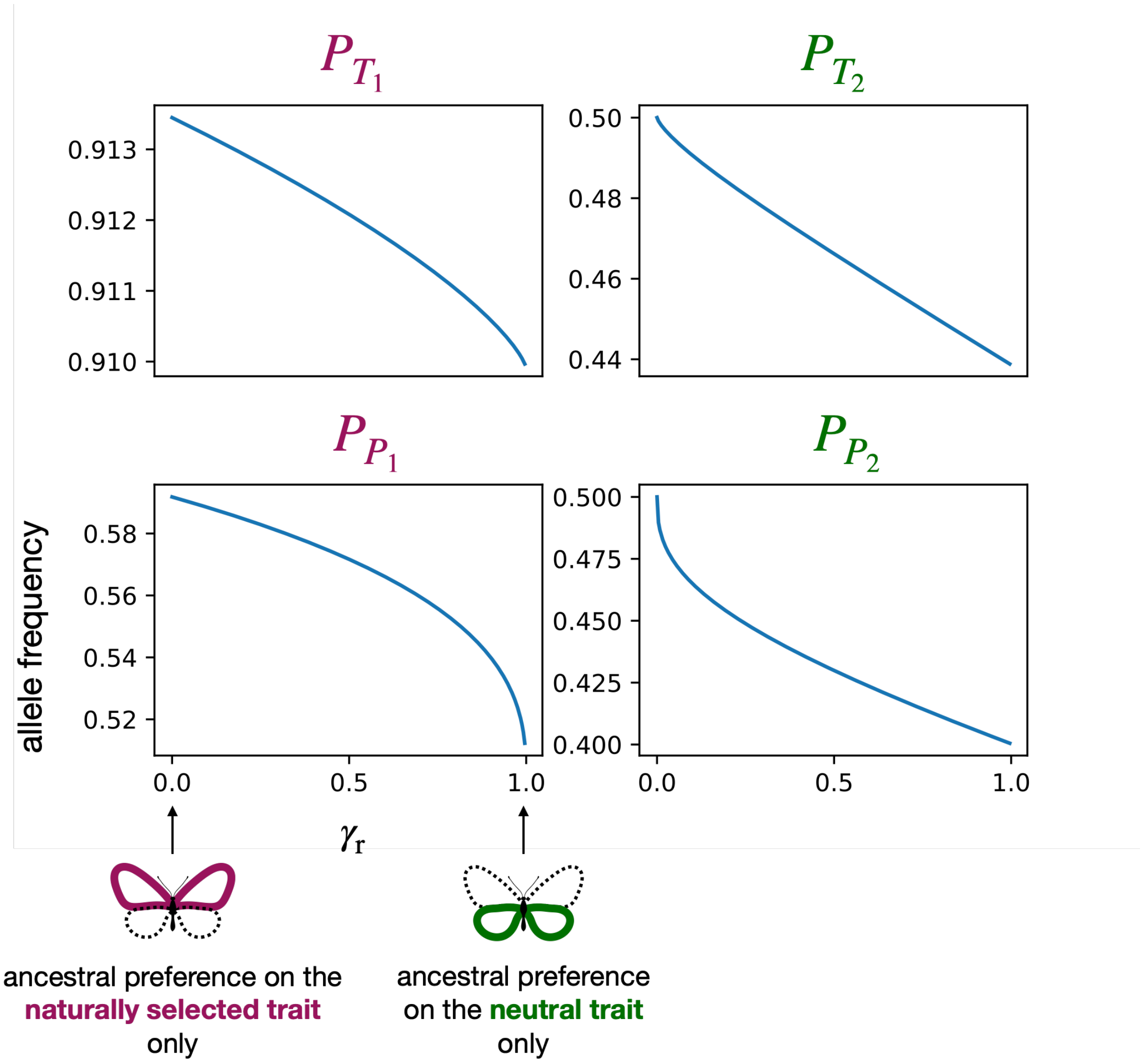

Figure A3: **Frequency of allele 1 at loci T_1, T_2, P_1 and P_2 depending on the resident *relative preference weighting* value (γ_r).** We assume reproductive interference ($c_{ri} = 0.001$), that trait T_1 is under natural selection ($s_1 = 0.05$) and trait T_2 is neutral ($s_2 = 0$).

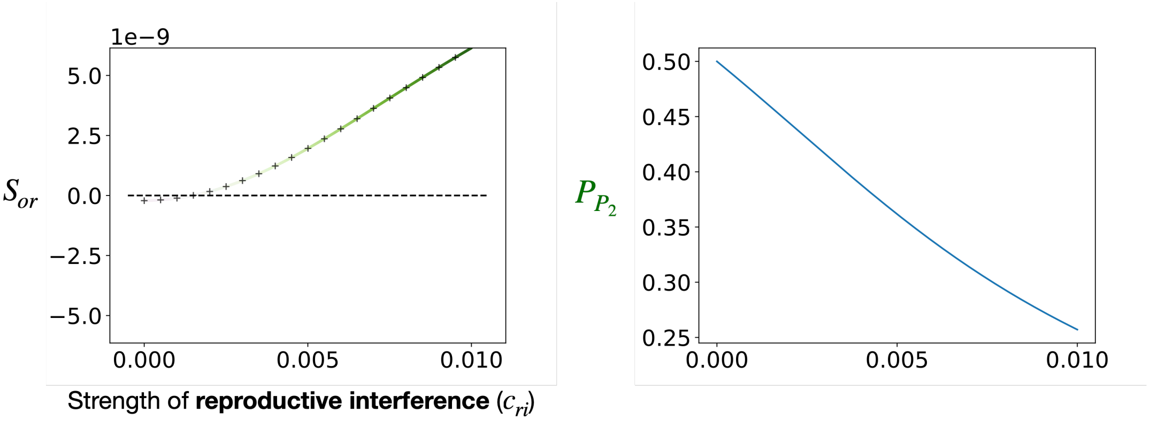

Figure A4: **Ancestral part of the selection gradient associated with offspring reproductive success (S_{or}) and frequency of allele 1 at locus P_2 (P_{P_2}) depending on the strength of reproductive interference (c_{ri}).** We assume ancestral preference targeting equally both traits ($\gamma_{\mathrm{t}_0} = 0.5$). We assume reproductive interference ($c_{\mathrm{ri}} = 0.0025$), that trait T_1 is under natural selection ($s_1 = 0.02$) and trait T_2 is neutral ($s_2 = 0$). When the line is green, offspring reproductive success promotes the evolution of preference towards the neutral selection T_2. The more intense the colour, the more intense the selection.

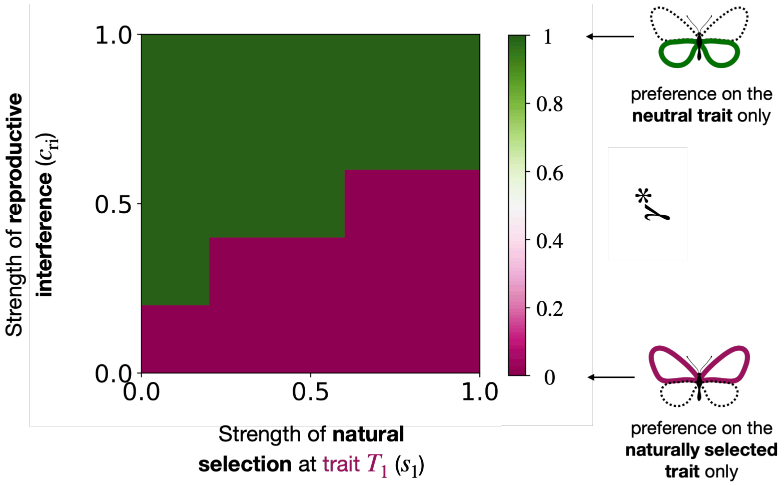

Figure A5: **Evolution of *relative preference weighting* towards a trait under selection T_1 or a neutral trait T_2 (γ^*), without the QLE assumptions, depending on the strength of natural selection acting on trait T_1 (s_1) and the strength of reproductive interference (c_{ri}) when T_2 is neutral ($s_2 = 0$). We assume $\rho = 1$, $c = 0.5$, $u_{T_1} = u_{T_2} = u_{P_1} = u_{P_2} = 0.2$.**

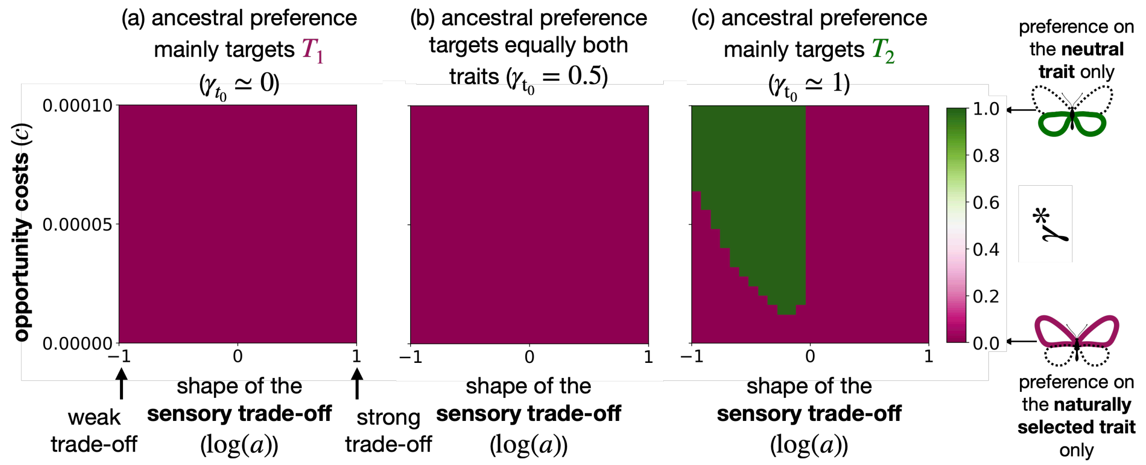

Figure A6: **Evolution of *relative preference weighting* towards a trait under selection T_1 or a neutral trait T_2 (γ^*) depending on the shape of the cognitive trade-off function (through the parameter a) and the cost of choosiness c for different ancestral preferences without species interactions $c_{\text{ri}} = 0$.** We assume ancestral preference targeting: (a) mainly trait T_1 ($\gamma_{t_0} = 0.01$), (b) equally both traits ($\gamma_{t_0} = 0.5$), (c) mainly trait T_2 ($\gamma_{t_0} = 0.99$). We assume that trait T_1 is under natural selection ($s_1 = 0.02$) and trait T_2 is neutral ($s_2 = 0$).

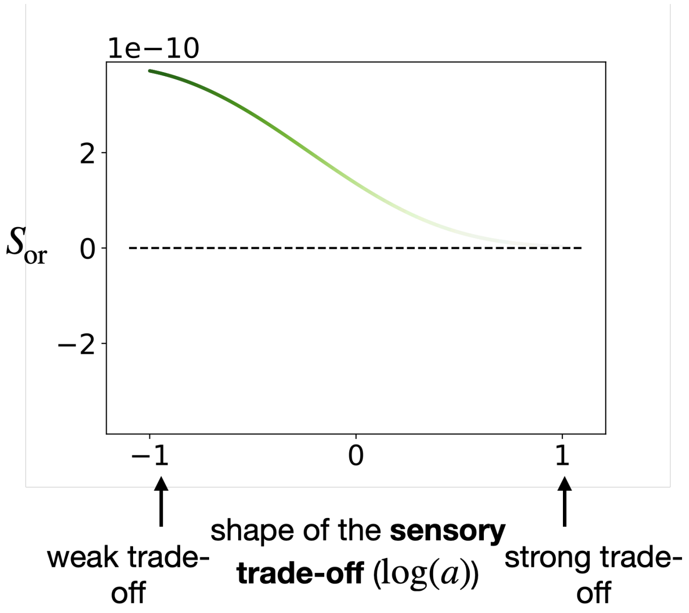

Figure A7: **Ancestral part of the selection gradient associated with offspring reproductive success (S_{or}) depending on the shape of the trade-off function (through the parameter a).** We assume ancestral preference targeting equally both traits ($\gamma_{t_0} = 0.5$). We assume reproductive interference ($c_{\text{ri}} = 0.025$), that trait T_1 is under natural selection ($s_1 = 0.02$) and trait T_2 is neutral ($s_2 = 0$). When the line is green, offspring reproductive success promotes the evolution of preference towards the neutral selection T_2. The more intense the colour, the more intense the selection.

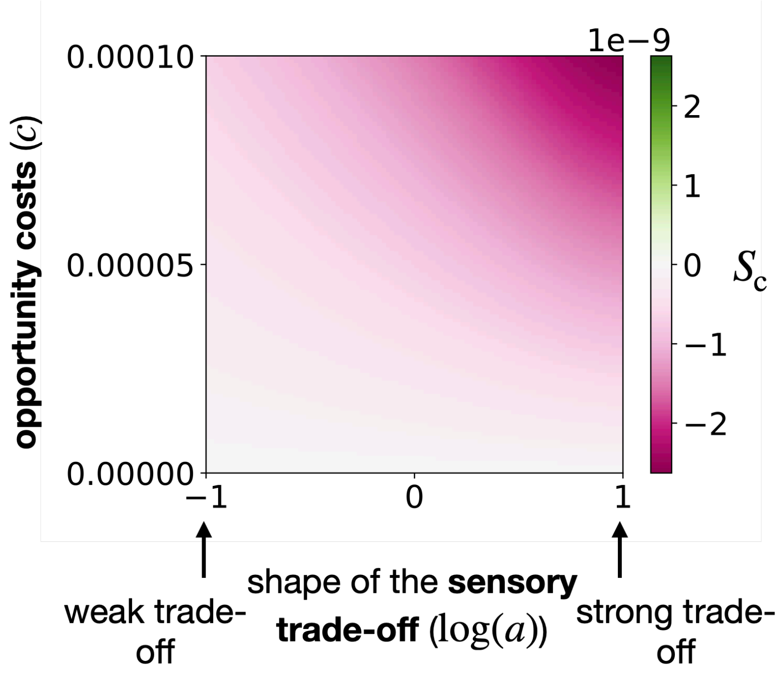

Figure A8: **Ancestral part of the selection gradient associated with cost of choosiness (S_c).** We investigate different shapes of the trade-off function (through the parameter a) and the cost of choosiness c. We assume ancestral preference targeting both traits equally ($\gamma_{t_0} = 0.5$). We assume reproductive interference ($c_{ri} = 0.025$), that trait T_1 is under natural selection ($s_1 = 0.02$) and trait T_2 is neutral ($s_2 = 0$). Purple area indicates that the cost of choosiness promotes the evolution of preference towards the trait under selection T_1.

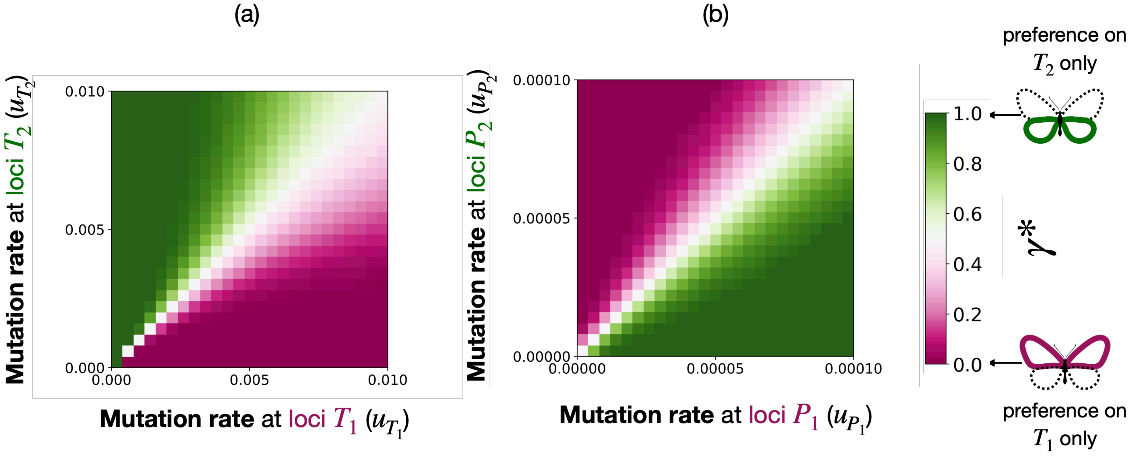

Figure A9: **Evolution of *relative preference weighting* towards traits T_1 or T_2 (γ^*), depending on (a) the mutation rates at loci T_1 and T_2 (u_{T_1} and u_{T_2}) and on (b) the mutation rates at loci P_1 and P_2 (u_{P_1} and u_{P_2}), without species interaction ($c_{\text{ri}} = 0$).** We assume that both traits are under natural selection ($s_1 = s_2 = 0.02$). When mutation rates at the two trait loci (T_1 and T_2) differ, the model predicts that female will prefer the traits associated with the highest mutation rate (see Figure A9). Mutations increase the number of males with a maladapted trait value. Such preference reduce mating with males with the maladapted trait value. When assuming that the mutation rate can differ at the loci P_1 and P_2, determining the preferred allele at trait T_1 and T_2 respectively, the model predicts that female will prefer the trait targeted by the preference locus with the lowest mutation rate (see Figure A9). Mutations at preference loci P_1 and P_2 indeed increase preference for the maladapted trait value, decreasing the likelihood of producing locally adapted offspring.
